# Evaluation of the efficacy of cephalosporin antibiotics sold in Kano, Nigeria, against clinical bacterial isolates

**DOI:** 10.1099/acmi.0.000837.v4

**Published:** 2025-07-14

**Authors:** Safiyya Atiku Ibrahim, Bashir Muhammad, Ismail Rabiu, Abdulazeez Muhammed

**Affiliations:** 1 Department of Microbiology, Faculty of Life Sciences, College of Natural and Pharmaceutical Sciences, Bayero University, P.M.B. 3011, Kano, Nigeria; 2 Department of Biotechnology, Delft University of Technology, Van der Maasweg 9, 2629 HZ Delft, Netherlands; 3 Department of Microbiology, School of Science and Information Technology, Skyline University Nigeria, Kano, Nigeria

**Keywords:** branded cephalosporins, *Escherichia coli*, Fourier transform infrared spectroscopy, Kano, resistant gene, Sabon Gari Market

## Abstract

**Introduction.** The evaluation of the efficacy of cephalosporin antibiotics sold in Kano, Nigeria, against clinical bacterial isolates is a timely and crucial public health concern. Cephalosporins are among the most widely used antibiotic classes globally due to their broad-spectrum activity and low toxicity. They play a vital role in the empirical treatment of infections involving both Gram-positive and Gram-negative bacteria. However, the increasing misuse and circulation of substandard or falsified antibiotics, especially in informal markets, threatens their therapeutic effectiveness.

**Gap statement.** In Nigeria, several formal and informal reports of substandard antibiotics and their unregulated sale in open markets, such as Sabon Gari in Kano, raise serious concerns about the quality of drug and their potential contribution to antimicrobial resistance (AMR). Cephalosporins sold in these markets are often sourced and stored under questionable conditions, which increases the risk of reduced potency or inefficacy. At the same time, AMR continues to rise globally, and in low and middle-income countries like Nigeria, poor-quality antibiotics can worsen treatment failure and drive resistance. Sabon Gari market is a major distribution centre for pharmaceuticals, yet research on its impact and extent of substandard drugs is lacking.

**Aim.** This study aims to assess the pharmaceutical quality of commonly sold cephalosporins purchased from drug distributors in Sabon Gari Market, Kano, by comparing their antimicrobial activity against a panel of clinical bacterial isolates (*Escherichia coli*, *Klebsiella pneumoniae*, *Staphylococcus aureus*, *Streptococcus pneumoniae*, *Pseudomonas aeruginosa* and *Salmonella* sp.) with that of standard reference antibiotics.

**Methodology.** Antimicrobial susceptibility pattern of the test isolates was determined by the disc diffusion method. Fourier transform infrared spectroscopy analysis was used to confirm the functional group of the active ingredients of all the antibiotics tested. Molecular identification of the resistant gene (CTX-M1) was carried out using PCR.

**Results.** Market survey (*n*=100) reveals that among drug distributors in Sabon Gari Market, Kano, cephalexin 61% (first generation); cefuroxime 72% (second generation); cefixime 68%, cefpodoxime 79%, ceftriaxone 63%, ceftazidime 70% and cefotaxime 45% (third generation); cefepime 84% (fourth generation) were the most commonly sold cephalosporins, with different brands and company names. These percentages represent the proportion of respondents who reported each antibiotic as one of their most frequently sold products. There is no significant difference between the branded antibiotics (drugs purchased in the market) and standard drugs. Exactly 20% of *E. coli* and *K. pneumoniae* were resistant, while 80% of *Staphylococcus aureus*, *Streptococcus pneumoniae*, *Pseudomonas* and *Salmonella* sp. were susceptible. The CTX-M1 resistant gene was identified in * E. coli* and *K. pneumoniae,* which further confirms their resistance to cefotaxime and ceftriaxone antibiotics.

**Conclusion.** Branded cephalosporins sold in Kano were chemically intact and structurally aligned with their respective formulations. No missing or anomalous functional groups were observed that would suggest substandard or counterfeit products, thus fit for human intake.

## Data Summary

All data are reported in the article and supplementary materials.

## Introduction

The primary rationale for this study is rooted in the growing global concern over the circulation of substandard antibiotics. Such antibiotics, often containing incorrect amounts of active ingredients or inferior quality compounds, can fail to effectively treat infections. The classification of antibiotics sold in Sabon Gari Market as ‘substandard’ in this study is based on existing reports and regulatory alerts rather than direct chemical content analysis. Several reports from the National Agency for Food and Drug Administration and Control (NAFDAC), government publications and investigative journalism have highlighted the circulation of substandard and falsified drugs in open markets across Nigeria, including Sabon Gari Market. This ineffectiveness can lead to the survival of drug-resistant bacteria, thereby exacerbating the problem of antibiotic resistance (AR). AR poses a significant threat to public health worldwide, as it can render common infections untreatable and increase the risk of severe illness and death. Sabon Gari Market, a major pharmaceutical distribution centre in Kano, Nigeria, has been reported both formally and informally to be a hotspot for the sale of substandard antibiotics [1,2]. Among these are the NAFDAC [2]. Despite its importance, there has been a noticeable gap in research specifically focusing on the quality of antibiotics sold in this market. Addressing this gap is crucial because identifying and understanding the prevalence of substandard antibiotics in such a key distribution hub can inform better regulatory and enforcement strategies, ultimately helping to mitigate the spread of AR. Therefore, the study seeks to explore the extent of this issue in Sabon Gari Market to inform public health interventions and policy decisions.

Sabon Gari Market in Nigeria was selected for this study due to multiple factors that make it an ideal candidate for investigating the prevalence of substandard antibiotics. Firstly, the market is a significant pharmaceutical distribution centre, supplying medications not only within Kano but also to various parts of Nigeria. Its extensive reach means that the quality of drugs sold here can have widespread implications for public health [3,4]. The circulation of substandard antibiotics clearly denotes that consumers might be purchasing medications that do not meet the necessary quality standards, potentially leading to ineffective treatments and contributing to the rise of antibiotic-resistant bacteria. Despite these alarming reports, there has been a lack of comprehensive research specifically targeting the impact and extent of substandard drug sales in this market. By focusing on Sabon Gari, this study aims to fill this research gap, providing valuable data on the quality of antibiotics available and the potential risks posed to public health.

Cephalosporins are a class of broad-spectrum antibiotics commonly used to treat a variety of bacterial infections. They are one of the most prescribed classes of antibiotics in the world [5]. Cephalosporins are classified by generation; in general, lower-generation cephalosporins have more Gram-positive activity, and higher-generation cephalosporins have more Gram-negative activity. The fourth-generation drug cefepime is the exception with Gram-positive activity equivalent to first-generation and Gram-negative activity equivalent to third-generation cephalosporins [6]. Third-generation cephalosporins are less active against Gram-positive cocci; they are much more active against enterobacteria and multiple-resistant bacteria. A major advantage of third-generation cephalosporins is that they are active against Gram-negative rods and are also useful in the management of hospital-acquired infections like bacteraemia and pneumonia.

The specific bacterial isolates under investigation include *Escherichia coli*, *Klebsiella pneumoniae*, *Staphylococcus aureus*, *Streptococcus pneumoniae*, *Pseudomonas aeruginosa* and *Salmonella* sp. These bacteria were chosen due to their clinical significance and the frequent occurrence of resistance among them [7]. Worldwide, antibiotic resistance threatens progress in health care, food production and ultimately life expectancy [8]. The world has not yet been able to find a way to collectively manage and control antibiotic resistance [9,10]. The problem is multi-sectoral and involves many complex challenges. Apart from the medical components, it has economic, ecological, sociological and developmental dimensions. Resistance to cephalosporins is creating serious health problems worldwide, especially in developing countries where bacterial infections contribute significantly to mortality and morbidities [9,11]. This study will further provide a comprehensive understanding of the quality and efficacy of cephalosporins sold in Sabon Gari Market. This information is crucial for developing strategies to combat the distribution of substandard antibiotics and addressing the issue of antibiotic resistance in Kano, Nigeria, and globally.

## Methods

### Study area

Kano is located in northern Nigeria. The state is bordered by the states of Jigawa to the north and east, Bauchi to the southeast, Kaduna to the southwest and Katsina to the northwest. According to the 2006 census figures from Nigeria, Kano State had a population totalling 9,401,288. Officially, Kano State is the most populous state in the country [12]. The popular Sabon Gari Market and Niger road, to be precise, is known for the sale of various pharmaceutical items including drugs. The market is popularly known for selling drugs both at wholesale and retail prices, and the majority of neighbouring states, such as Jigawa, Katsina and Sokoto, as well as some neighbouring countries like Niger Republic, source pharmaceutical products from the market.

### Survey of commonly sold brands of cephalosporins

A total of 100 questionnaires (Appendix II) were distributed among drug distributors at Niger Street Sabon Gari Market Kano, Nigeria. This was done in order to have knowledge about the most commonly sold cephalosporin antibiotics in the study location. The survey results provide valuable insights into the cephalosporin antibiotic market in Sabon Gari, Kano. Each generation of cephalosporins has its most commonly sold antibiotic, indicating diverse usage patterns and preferences among drug distributors and medical practitioners.

### Collection of antibiotics and preparation of filter paper disc

After distribution of the questionnaire, antibiotics were bought from drug distributors at Niger Street Sabon Gari Market, Kano State. The antibiotics bought include the first-generation to fourth-generation cephalosporin antibiotics. The first generation was cephalexin; the second generation was cefuroxime. Among the third generation were cefotaxime, cefpodoxime, cefixime, ceftazidime and ceftriaxone. The fourth generation was cefepime. The standard disc of all the antibiotic generations was purchased from Biorapid Diagnostic Centre, Zaria, Kaduna. For each antibiotic, the contents were aseptically reconstituted with DMSO to achieve the standard disc concentration (typically 30 µg/disc, depending on the specific cephalosporin). Whatman no. 1 filter paper discs (6 mm diameter) were then impregnated with 20 µl of each antibiotic solution, air-dried under sterile conditions and stored in sealed, sterile containers at 4 °C until use. All procedures followed the standard guidelines as adapted from the Clinical and Laboratory Standards Institute (CLSI) protocols for antimicrobial disc preparation.

### Collection of bacterial samples

Ten clinical bacterial isolates (for each organism) were collected from the Medical Microbiology Laboratory, Murtala Muhammad Specialist Hospital, Kano. This makes a total of 60 isolates. The isolates (*E. coli*, *K. pneumoniae*, *Staphylococcus aureus*, *Streptococcus pneumoniae*, *Pseudomonas aeruginosa* and *Salmonella* spp.) were obtained from stool samples of patients presented at the hospital. Selection criteria included isolate viability, confirmed identification and relevance to common infection presentations in the hospital setting. The rationale for collecting up to ten isolates per species was to ensure diversity and allow for the inclusion of strains associated with different clinical conditions. This approach provided a broader representation of bacterial infections in the study area. Further analysis was conducted at the Microbiology laboratory, Bayero University, Kano.

### Confirmation of the bacterial isolates

Isolates were sub-cultured on Mannitol salt agar (*Staphylococcus aureus*), MacConkey (*Pseudomonas*, *E. coli* and *K. pneumoniae*), blood base (*Streptococcus pneumoniae*) and *Salmonella Shigella* agar (*Salmonella* spp*.*) and incubated at 37 °C for 24 h. The bacterial isolates were identified using the active pharmaceutical ingredient (API) system and were characterized by examining the colonial morphology through Gram reaction, followed by biochemical tests such as sugar fermentation (lactose, sucrose and glucose), catalase and oxidase tests [13].

#### Sugar fermentation (triple sugar iron) test

The triple sugar iron (TSI) test was used to assess bacterial ability to ferment sugars and produce hydrogen sulphide. Following the manufacturer’s instructions, TSI agar was prepared and solidified in a slant. Bacterial samples were inoculated, stabbed into the media and incubated for 18–24 h [13]. Red slant/yellow butt indicates glucose fermentation. Yellow slant and butt indicate lactose or sucrose fermentation. Agar lift at the bottom indicates gas production, while hydrogen sulphide production is inferred following the formation of a dark colour in the middle of each colony.

#### Biochemical test

##### Oxidase test

A piece of filter paper was placed in a clean petri dish, and two to three drops of freshly prepared oxidase reagent were added. A colony was then smeared on the reagent-impregnated paper using a glass rod. A positive result is indicated by a blue to purple colour appearing within a few seconds [13].

##### Indole test

Colonies were picked using a glass rod and immersed in a test tube containing 5 ml of peptone water and then incubated for 24 h. After incubation, 5 drops of Kovac’s reagent were added to the culture broth, which was gently shaken. A colour change from yellow to red indicated a positive result [13].

##### Coagulase test

A drop of distilled water was placed on two separate slides. A colony of the test organism was emulsified in each drop to create two thick suspensions. A loopful of plasma was added to one suspension and mixed gently. Clumping of the organisms within 10 s indicated a positive result for coagulase. The second suspension, without plasma, was used to differentiate true coagulase clumping from any granular appearance of the organisms [13].

##### Catalase test (slant method)

Two millilitres of hydrogen peroxide solution were poured into a test tube. Using a sterile wooden stick or glass rod (not a nichrome wire loop), several colonies of the test organism were added to the hydrogen peroxide solution. Immediate bubbling indicated the presence of catalase [13].

### Antimicrobial susceptibility test of the standard disc

This was done using the Kirby–Bauer disc diffusion method with reference to the CLSI performance guideline for antimicrobial susceptibility testing [14]. Bacteria were grown in the nutrient broth at 37 °C overnight. The suspensions were visually adjusted with normal saline to match the 0.5 MacFarland (1.5×108 c.f.u. ml^−1^) turbidity standard. Inoculated plates were left to stand for at least 3 min before standard antibiotic discs, cephalexin (30 µg), cefuroxime (30 µg), cefixime (30 µg), cefpodoxime (30 µg), ceftazidime (30 µg), ceftriaxone (30 µg), cefotaxime (30 µg) and cefepime (30 µg) were placed. This was followed by overnight incubation at 37 °C. The diameter zone of inhibition was measured and interpreted according to the CLSI guideline.

### Fourier transform infrared spectroscopy analysis

The purchased antibiotics were subjected to Fourier transform infrared spectroscopy (FT-IR) analysis in order to confirm the functional group additives or contaminants in the sampled antibiotics [15,16].

#### Testing process

The IR beam was disinfected before and after each antibiotic was removed. The antibiotics were placed in the FT-IR spectrometer, which directed beams of IR at the antibiotics, and measured how much of the beam, and at which frequencies, the antibiotics absorbed the infrared light. The spectra produced a profile of the sample, a distinctive molecular fingerprint that was used to screen antibiotics for different components [15,16].

### Genomic DNA isolation

The procedure was carried out according to the manufacturer’s instructions. Exactly 2 ml of phosphate buffer was added to the bijou bottles containing the isolates. The washed isolates were then poured into an Eppendorf tube and centrifuged at 14,000 r.p.m. for 5 min. This was followed by the addition of 400 µl of buffer and 25 µl of protein K to the tube containing the isolate. Following an hour of incubation at a heat block with vortex every 20 min [17].

This was followed by the addition of phenol chloroform (400 µl), vortexed and centrifuged at 1,300 r.p.m. for 10 min. The upper layer was collected, and 100% ethanol and 40 µl of 3M sodium acetate were added and incubated at −20 °C overnight. Finally, 70% ethanol was added and centrifuged, and the upper layers were discarded, while the contents were allowed to air dry [18].

### PCR method for detection of the CTX-M1 gene

Using the primer sequence (CTX-M1: F: CTT CCA GAA GAA GGA ATC, R: CCG TTT CCG CTA TTA CAA, at 800 bp), the amplification was carried out according to the method described by Bush and Bradford [17] and Yahya *et al*. [19]. Pre-denaturation step, denaturation and annealing of primers were achieved at a temperature of 95 °C for 5 min, 94 °C for 30 s and 55 °C for 30 s, respectively. This was followed by extension at 72 °C for 10 min. Following the completion of the amplification processes, the DNA band of molecular weight 800 bp in compared with the molecular weight marker. DNA amplicons were visualized using a gel imaging system.

## Result and Discussion

Sabon Gari Market Kano is renowned as one of the largest markets that contains different drug pharmaceutical distributors, selling different kinds of drugs including antibiotics. As such, different patients patronize this market for their drug needs. This informed the choice for the selection of this market for the identification of the most commonly used antibiotics belonging to different generations of cephalosporin antibiotics (Table 1). The market survey (conducted using questionnaire, Appendix II) presented in Table 1 showed that among drug distributors in Sabon Gari Market, cephalexin (first generation); cefuroxime (second generation); cefixime, cefpodoxime, ceftriaxone, ceftazidime and cefotaxime (third generation); and cefepime (fourth generation) were the most commonly sold brand of cephalosporins in Sabon Gari Market, Kano State, Nigeria. The choice of cephalosporin antibiotics in Sabon Gari Market is influenced by factors such as cost, efficacy, availability and medical prescription patterns. The findings emphasize the need for continuous monitoring and regulation of antibiotic sales, particularly cephalosporins, to ensure their quality and effectiveness and, by extension, to safeguard public health against the threat of antibiotic resistance. However, it is important to acknowledge that this study focused solely on cephalosporins. Since a variety of antibiotic classes are sold in Sabon Gari Market, generalizing the efficacy of all antibiotics based on cephalosporins alone presents a limitation. Therefore, future studies are recommended to include a broader range of antibiotics to provide a more comprehensive assessment of antibiotic quality and resistance trends in the market. Of all the eight different antibiotics employed in this study, the third- and fourth-generation cephalosporin antibiotics were observed to have the highest number of the most commonly sold antibiotics, with ceftriaxone, cefepime, ceftazidime and cefpodoxime having 83%, 81%, 80% and 79% respectively. This might be linked with the fact that the third-generation cephalosporins are more effective against Gram-negative bacteria than the first and second generations [1]. They are usually effective against bacteria that may be resistant to previous generations of cephalosporins [20].

**Table 1.** Identification of the most commonly sold antibiotics belonging to different cephalosporin generations in the market (Drug Dealers Sabon Gari Market Kano) n = 100

Generation	Cephalosporin antibiotic (%)	Brand name	Company name
**First generation**	Cephalexin (51)	Daxbia, kefkex	Cefamor
**Second generation**	Cefuroxime (62)	Ceftin, zimcrf	Tambac
**Third generation**	Cefixime (68)	Suprax	Ocefix
	Cefpodoxime (79)	Generic	Tambac
	Ceftriaxone (83)	Rocephin	Zoxon
	Ceftazidime (80)	Fortez, tazicef	Bioszime
	Cefotaxime (52)	—	—
**Fourth Generation**	Cefepime (81)	Maxipime	Supasil

More so, the high use of these third- and fourth-generation cephalosporin antibiotics could be due to their effectiveness in treating infections such as lower respiratory infections, urinary tract infections, intra-abdominal infections, gynaecological infections, sepsis, joints and skin and soft tissue infections of which different patients are diagnosed so frequently [21]. In addition, D’Errico *et al.* [1] reported that fewer complications such as side effects have been associated with the use of these classes of antibiotics.

A total of 60 isolates (10 of each) were reconfirmed, having *Staphylococcus aureus* and *Streptococcus pneumoniae* and *P. aeruginosa*, *Salmonella*, *K. pneumoniae* and *E. coli* as Gram-positive and Gram-negative isolates, respectively (Table 2). Upon comparison with published literature, the results agree with the findings of Cheesbrough [22] and Chika *et al.* [23]. All reconfirmed isolates were tested with both the standard and prepared antibiotic discs impregnated with all the commonly identified cephalosporin antibiotics (cephalexin, cefuroxime, cefixime, cefpodoxime, ceftriaxone, ceftazidime, cefotaxime and cefepime) at a disc potency concentration of 30 µg/disc.

**Table 2.** Biochemical, Gram staining and microscopic characteristics of test isolates

Test	*K. pneumoniae*	*E. coli*	*Staphylococcus aureus*	*Streptococcus pneumoniae*	*P. aeruginosa*	*Salmonella*
**Biochemical test**						
Indole	--ve	+ve	--ve	+ve	--ve	--ve
Gas production	+ve	+ve	--ve	--ve	+ve	+ve
H_2_S production	--ve	--ve	--ve	--ve	--ve	+ve
Coagulase	--ve	--ve	+ve	--ve	--ve	--ve
Motility	--ve	+ve	--ve	--ve	+ve	+ve
Oxidase	--ve	--ve	--ve	--ve	+ve	--ve
Glucose	+ve	+ve	+ve	+ve	--ve	+ve
Lactose	+ve	+ve	+ve	+ve	--ve	--ve
Catalase	+ve	+ve	+ve	--ve	+ve	+ve
**Microscopy**						
Morphology	Rod	Rod	Cocci	Diplococci	Rod/cocci	Rod
Gram reaction	Gram -ve	Gram -ve	+ve	+ve	--ve	--ve

H2S, hydrogen sulphide; --ve, negative; +ve, positive.

Upon comparing the activities of each of the standard and prepared cephalosporin antibiotics using a t-test, all the antibiotics tested against the test isolates [*E. coli* (Table 3), *K. pneumoniae* (Table 4), *Staphylococcus aureus* (Table 5), *Streptococcus pneumoniae* (Table 6), *P. aeruginosa* (Table 7) and *Salmonella* (Table 8)] recorded no statistically significant differences in their activity, *P*>0.05. The higher activity recorded by the antibiotics against the test isolates is in line with the report of Deborah *et al*. [24], Abdulfatai *et al*. [25] and Santosh *et al*. [26], who reported varying levels of activity among different antibiotics tested against *E. coli*, *Salmonella* and *Klebsiella* spp., with the highest activity to cephalosporin antibiotics. The lower activity exhibited by the test isolate on the prepared antibiotic discs might be due to the disc potency, as the discs have not been soaked to the required level of the antibiotic. Substandard drugs may result from poor manufacturing practices, inadequate storage conditions (especially in high-temperature environments like open drug markets) or degradation over time. These factors can significantly reduce the potency of the API, even if the correct compound is present [1,27]. This potential substandard quality may explain the reduced efficacy observed in susceptibility testing compared to standard reference drugs. To strengthen our interpretation, future studies could incorporate quantitative assays such as HPLC or LC-MS to determine the actual API concentration and confirm whether the observed reduction in activity is due to poor disc preparation or substandard drug quality. For the current study, we acknowledge both as possible contributors and have expanded our discussion accordingly to reflect this balanced view.

**Table 3.** Susceptibility of *E. coli* to commonly prescribed cephalosporins (standard and laboratory prepared disc)

	CFL(*P*=0.91)		CXM(*P*=0.95)		CFM(*P*=0.99)		CPD(*P*=0.95)		CRO(*P*=0.98)		CAZ(*P*=0.99)		CTX(*P*=1.0)		CEF(*P*=0.99)	
	SD	PD	SD	PD	SD	PD	SD	PD	SD	PD	SD	PD	SD	PD	SD	PD
E1	23±0.1	22±0.1	19±0.1	18±0.1	20±0.1	19±0.1	18±0.0	17*±0.1	22±0.1	19*±0.1	23±0.1	21±0.1	27±0.1	24±0.1	25±0.1	23±0.1
E2	20±0.1	19±0.2	20±0.2	19±0.1	21±0.2	19±0.3	19±0.6	18±0.0	25±0.1	23±0.3	22±0.1	20±0.1	23*±0.1	22*±0.3	26±0.3	23±0.3
E3	22±0.3	22±0.2	19±0.4	18±0.2	19±0.1	17±0.2	20±0.5	19±0.5	24±0.5	22±0.1	23±0.1	22±0.0	24±0.1	23*±0.0	28±0.2	25±0.2
E4	14*±0.2	14*±0.2	13*±0.5	11*±0.3	17*±0.1	15*±0.1	17*±0.5	13*±0.1	10*±0.5	09*±0.3	20±0.2	17*±0.0	14*±0.2	10*±0.0	21±0.3	20±0.1
E5	23±0.5	21±0.3	20±0.5	19±0.1	20±0.2	19±0.1	20±0.5	18±0.1	22±0.5	20±0.2	22±0.5	21±0.1	25±0.2	25±0.5	27±0.4	26±0.1
E6	22±0.1	21±0.4	18±0.	17±0.1	22±0.1	20±0.3	20±0.5	19±0.3	22±0.1	19*±0.2	23±0.7	22±0.2	24±0.3	23*±0.4	25±0.1	23±0.1
E7	15±0.2	15±0.3	14*±0.0	12*±0.2	17*±0.	15*±0.3	18±0.1	15*±0.3	19*±0.0	16*±0.3	18±0.5	17*±0.1	13*±0.3	10*±0.2	24±0.1	23±0.3
E8	22±0.2	21±0.1	19±0.	19±0.2	20±0.4	19±0.5	19±0.1	18±0.1	24±0.1	23±0.4	22±0.2	21±0.1	26±0.2	24±0.1	27±0.7	26±0.4
E9	20±0.4	19±0.1	20±0.	20±0.3	20±0.3	18±0.5	20±0.3	19±0.3	24±0.0	23±0.1	23±0.3	22±0.3	25±0.2	24±0.0	25±0.0	24±0.6
E10	24±0.5	22±0.1	21±0.	21±0.3	20±0.2	18±0.5	20±0.1	18±0.1	24±0.0	23±0.0	20±0.3	17*±0.1	25±0.1	24±0.0	25±0.0	24±0.0
R	1	1	2	2	2	2	1	2	2	3	0	2	3	5	0	0
S	9	9	8	8	8	8	9	8	8	7	10	8	7	5	10	10

*Resistance.

CAZ, Ceftazidime; CFL, Cephalexin; CFM, Cefixime; CPD, Cefpodoxime; CRO, Ceftriaxone; CTX, Cefotaxime; CXM, Cefuroxime; E, *E. coli*; FEP, Cefepime; PD, prepared disc; R, resistant; RP, resistant to prepared antibiotics; RS, resistant to standard antibiotics; S, susceptible; SD, standard disc.

**Table 4.** Susceptibility of *K. pneumoniae* to commonly prescribed cephalosporins (standard and laboratory prepared disc)

	CFL(*P*=1.0)		CXM(*P*=0.99)		CFM(*P*=1.0)		CPD(*P*=0.98)		CRO(*P*=0.99)		CAZ(*P*=1.1)		CTX(*P*=1.01)		FEP(*P*=0.99)	
	SD	PD	SD	PD	SD	PD	SD	PD	SD	PD	SD	PD	SD	PD	SD	PD
K1	22±0.1	21±0.1	20±0.5	19±0.1	20±0.3	19±0.5	22±0.0	19±0.3	21±0.1	20±0.1	23±0.0	20±0.3	23±0.2	22*±0.1	25±0.3	23±0.5
K2	23±0.1	22±0.2	20±0.1	19±0.1	20±0.2	19±0.3	21±0.	19±0.1	23±0.2	21±0.3	20±0.0	17*±0.0	24±0.2	23±0.2	26±0.2	25±0.3.
K3	14*±0.1	13*±0.3	15±0.1	14*±0.2	17*±0.3	15*±0.1	18±0.1	15*±0.3	15*±0.2	14*±0.5	18±0.5	18±0.0	13*±0.1	12*±0.5	20±0.2	20±0.3
K4	22±0.2	21±0.1	18±0.2	15±0.2	18±0.2	18±0.1	20±0.2	18±0.2	20±0.1	20±0.1	20±0.1	17*±0.2	24±0.1	23±0.1	27±0.2	26±0.2
K5	23±0.0	21±0.3	18±0.2	16±0.2	19±0.2	18±0.5	21±0.7	20±0.1	23±0.1	22±0.0	23±0.2	21±0.0	24±0.2	23±0.2	28±0.1	25±0.2
K6	21±0.1	20±0.2	18±0.1	16±0.2	18±0.2	18±0.3	22±0.2	19±0.3	21±0.3	20±0.1	22±0.1	20±0.2	22*±0.1	22*±0.3	28±0.2	25±0.2
K7	15±0.1	13*±0.1	13*±0.2	13*±0.2	17*.3	15*±0.3	18±0.5	17*±0.2	19*±0.3	19*±0.3	18*±0.3	15*±0.1	15*±0.1	13*±0.3	23±0.3	21±0.1
K8	20±0.2	19±0.3	19±0.1	18±0.0	19±0.5	18±0.3	20±0.1	18±0.5	21±0.5	20±0.3	23±0.0	22±0.0	25±0.1	24±0.2	27±0.2	25±0.3
K9	20±0.2	18±0.0	20±0.1	18±0.2	20±0.2	19±0.1	20±0.3	18±0.3	23±0.3	21±0.2	22±0.1	20±0.1	24±0.2	23±0.2	25±0.1	24±0.1
K10	22±0.1	21±0.1	19±0.1	17±0.2	19±0.2	18±0.2	20±0.1	19±0.1	22±0.1	21±0.3	21±0.1	20±0.1	24±0.3	23±0.1	28±0.2	25±0.1
R	0	2	1	2	2	2	0	2	2	4	1	3	2	4	0	0
S	10	8	9	8	10	10	10	9	9	6	10	7	6	2	10	10

*Resistance.

CAZ, Ceftazidime; CFL, Cephalexin; CFM, Cefixime; CPD, Cefpodoxime; CRO, Ceftriaxone; CTX, Cefotaxime; CXM, Cefuroxime; FEP, Cefepime; K, *K. pneumoniae*; PD, prepared disc; R, resistant; RP, resistant to prepared antibiotics; RS, resistant to standard antibiotics; S, susceptible; SD, standard disc.

**Table 5.** Susceptibility of *Staphylococcus aureus* to commonly prescribed cephalosporins (standard and laboratory prepared disc)

	CFL(*P*=0.99)		CXM(*P*=0.99)		CFM(*P*=0.99)		CPD(*P*=0.99)		CRO(*P*=0.99)		CAZ(*P*=0.89)		CTX(*P*=1.0)		FEP(*P*=0.99)	
	SD	PD	SD	PD	SD	PD	SD	PD	SD	PD	SD	PD	SD	PD	SD	PD
S1	20±0.2	19±0.1	18±0.0	17±0.0	21±0.2	20±0.2	21±0.2	20±0.3	22±0.3	21±0.4	21±0.0	18±0.5	24±0.4	23*±0.3	27±0.4	25±0.4
S2	19±0.2	17±0.1	17±0.1	16±0.0	20±0.1	18±0.1	22±0.3	21±0.3	20±0.2	19*±0.2	20±0.1	17*±0.0	25±0.3	24±0.2	26±0.3	24±0.1
S3	18±0.3	16±0.1	18±0.0	17±0.0	19±0.2	18±0.1	21±0.1	20±0.3	22±0.1	21±0.3	21±0.4	18±0.1	24±0.1	23*±0.7	27±0.1	25±0.2
S4	18±0.2	16±0.1	19±0.5	18±0.0	20±0.2	18±0.2	20±0.2	18±0.3	21±0.1	19*±0.5	21±0.5	19±0.3	25±0.4	23*±0.5	28±0.0	27±0.5
S5	19±0.2	17±0.1	17±0.2	15±0.1	22±0.1	20±0.1	22±0.3	20±0.2	20±0.5	18*±0.4	22±0.5	20±0.1	26±0.5	25±0.5	26±0.1	24±0.0
S6	20±0.3	19±0.1	19±0.3	18±0.5	19±0.8	18±0.0	22±0.0	21±0.1	23±0.5	22±0.1	22±0.1	20±0.2	24±0.5	24±0.4	28±0.4	27±0.1
S7	18±0.5	17±0.2	16±0.1	14*±0.1	20±0.3	18±0.3	21±0.3	20±0.4	23±0.0	22±0.1	20±0.3	17*±0.1	25±0.	24±0.1	26±0.3	23±0.4
S8	19±0.1	17±0.2	18±0.3	17±0.1	20±0.2	18±0.5	20±0.5	19±0.1	23±0.3	22±0.4	21±0.1	19±0.5	26±0.5	25±0.7	27±0.2	25±0.5
S9	21±0.6	19±0.5	18±0.0	17±0.0	19±0.7	18±0.5	22±0.1	21±0.1	21±0.1	20±0.5	21±0.2	18±0.2	25±0.5	24±0.5	28±0.4	27±0.5
S10	21±0.1	19±0.3	17±0.1	16±0.2	19±0.1	17*±0.4	21±0.5	20±0.0	23±0.0	21±0.3	21±0.3	18±0.1	25±0.2	24±0.4	26±0.1	24±0.3
R	0	0	0	1	0	1	0	0	0	3	0	2	0	3	0	0
Sp	10	10	10	9	10	9	10	10	10	7	10	8	10	7	10	10

*Resistance.

CAZ, Ceftazidime; CFL, Cephalexin; CFM, Cefixime; CPD, Cefpodoxime; CRO, Ceftriaxone; CTX, Cefotaxime; CXM, Cefuroxime; FEP, Cefepime; PD, prepared disc; R, resistant; RP, resistant to prepared antibiotics; RS, resistant to standard antibiotics; *S. aureus*, *Staphylococcus aureus*; SD, standard disc; Sp, susceptible.

**Table 6.** Susceptibility of *Streptococcus pneumoniae* to commonly prescribed cephalosporins (standard and laboratory prepared disc)

	CFL(*P*=0.92)		CXM(*P*=0.99)		CFM(*P*=1.0)		CPD(*P*=1.0)		CRO(*P*=0.99)		CAZ(*P*=0.93)		CTX(*P*=0.99)		FEP(*P*=1.0)	
	SD	PD	SD	PD	SD	PD	SD	PD	SD	PD	SD	PD	SD	PD	SD	PD
**Sp1**	20±0.1	17±0.3	21±0.1	20±0.1	19±0.3	18±0.1	21±0.7	20±0.0	25±0.1	24±0.2	22±0.5	20±0.1	25±0.0	24±0.1	25±0.1	24±0.0
**Sp2**	19±0.1	16±0.1	20±0.3	19±0.1	19±0.2	18±0.5	24±0.2	23±0.0	26±0.4	25±0.1	21±0.1	18±0.1	23*±0.5	22*±0.3	26±0.0	25±0.1
**Sp3**	18±0.3	15±0.2	20±0.3	19±0.3	19±0.3	18±0.5	23±0.3	22±0.1	24±0.5	23±0.2	20±0.1	17*±0.2	25±0.1	24±0.2	26±0.1	25±0.0
**Sp4**	19±0.0	17±0.3	21±0.5	19±0.4	18±0.3	16*±0.2	22±0.1	20±0.9	22±0.1	19*±0.5	22±0.1	20±0.1	25±0.1	24±0.1	25±0.1	24±0.0
**Sp5**	20±0.0	17±0.1	19±0.1	17±0.1	20±0.7	19±0.2	23±0.5	22±0.2	23±0.2	22±0.6	21±0.3	18±0.1	25±0.2	24±0.1	27±0.1	26±0.1
**Sp6**	19±0.1	17±0.5	18±0.2	17±0.3	18±0.1	17*±0.0	24±0.1	23±0.3	24±0.2	23±0.2	21±0.2	19±0.3	24±0.1	22*±0.5	27±0.2	26±0.5
**Sp7**	21±0.0	18±0.1	18±0.3	17±0.4	20±0.1	19±0.2	24±0.4	23±0.2	25±0.2	24±0.1	20±0.1	17*±0.4	24±0.0	22*±0.5	25±0.1	24±0.5
**Sp8**	21±0.07	19±0.3	20±0.5	18±0.3	18±0.2	18±0.3	21±0.1	20±0.4	26±0.1	25±0.1	24±0.0	22±0.3	23*±0.0	20*±0.7	26±0.4	25±0.1
**Sp9**	20±0.0	18±0.2	19±0.2	17±0.2	20±0.1	18±0.2	22±0.5	20±0.1	23±0.3	22±0.2	22±0.0	20±0.3	25±0.1	24±0.1	27±0.1	26±0.4
**Sp10**	20±0.1	18±0.2	21±0.2	20±0.1	20±0.3	19±0.5	23±0.5	23±0.1	22±0.2	19*±0.1	21±0.2	18±0.4	25±0.5	24±0.1	27±0.1	26±0.7
**R**	0	0	0	0	0	2	0	0	0	2	0	2	2	4	0	0
**S**	10	10	10	10	10	8	10	10	10	8	10	8	8	6	10	10

*Resistance.

CAZ, Ceftazidime; CFL, Cephalexin; CFM, Cefixime; CPD, Cefpodoxime; CRO, Ceftriaxone; CTX, Cefotaxime; CXM, Cefuroxime; FEP, Cefepime; PD, prepared disc; R, resistant; RP, resistant to prepared antibiotics; RS, resistant to standard antibiotics; S, susceptible; SD, standard disc; Sp, *S. pneumoniae*.

**Table 7.** Susceptibility of *P. aeruginosa* to commonly prescribed cephalosporins (standard and laboratory prepared disc)

	CFL (*P*<0.05)		CXM (*P*<0.05)		CFM (*P*<0.05)		CPD (*P*<0.05)		CRO (*P*<0.05)		CAZ (*P*<0.05)		CTX (*P*<0.05)		FEP (*P*>0.05)	
	SD	PD	SD	PD	SD	PD	SD	PD	SD	PD	SD	PD	SD	PD	SD	PD
**P1**	20±	17±	19±	18±	20±	19±	24±	23±	20±	19±0*	23±	22±0	21±0*.	20±*	27±	26±
	0.1	0.1	0.1	0.1	0.2	0.1	0.1	0.5	0.2	0.5	0.2	0.0	1	0.1	0.2	0.0
**P2**	18±	16±	20±	19±	22±	21±	23±	22±	21±	20±0	22±	20±0	21±0*.	20±*	26±	25±
	0.1	0.0	0.2	0.2	0.2	0.1	0.5	0.1	0.1	0.5	0.3	0.1	7	0.0	0.1	0.1
**P3**	18±	16±	20±	19±	21±	19±	22±	21±	22±	21±0	21±	20±0	23±0*	22±*	28±	27±
	0.4	0.0	0.1	0.1	0.1	0.2	0.1	0.3	0.4	0.1	0.3	0.3	1	0.1	0.2	0.1
**P4**	20±	17±	20±	19±	20±	19±	21±	20±	20±	19±0*	21±	20±0	23±0*	22±*	25±	25±
	0.1	0.0	0.3	0.1	0.2	0.3	0.11	0.1	0.1	0.3	0.1	0.3	0	0.3	0.1	0.0
**P5**	19±	18±	19±	18±	21±	18±	22±	21±	22±	21±0	21±	19±0	23±0*	22±*	25±	25±
	0.1	0.4	0.3	0.2	0.1	0.2	0.3	0.2	0.2	0.1	0.3	0.1	0	0.5	0.2	0.1
**P6**	20±	17±	18±	17±	22±	21±	23±	22±	22±	21±0	23±	22±0	22±0*	20±*	26±	25±
	0.2	0.2	0.1	0.3	0.5	0.1	0.3	0.1	0.1	0.2	0.1	0.3	2	0.1	0.2	0.2
**P7**	18±	16±	18±	17±	20±	19±	23±	22±	21±	20±0	23±	22±0	22±0*	20±*	27±	26±
	0.1	0.2	0.0	0.2	0.1	0.3	0.1	0.3	0.2	0.3	0.4	0.3	2	0.5	0.4	0.1
**P8**	19±	18±	18±	17±	22±	21±	23±	22±	20±	19±0*	24±	23±0	22±0*	21±*	25±	24±
	0.1	0.2	0.0	0.2	0.2	0.1	0.1	0.1	0.5	0.1	0.6	0.2	2	0.0	0.2	0.3
**P9**	19±	18±	20±	18±	20±	19±	21±	20±	20±	19±0	24±	22±0	21±0*	20±*	28±	27±
	0.3	0.1	0.2	0.1	0.2	0.2	0.3	0.2	0.5	0.1	0.4	0.1	1	0.1	0.3	0.1
**P1**	19±	18±	19±	18±	21±	19±	21±	20±	22±	21±0	22±	20±0	22±0*	21±*	25±	24±
**0**	0.1	0.4	0.3	0.5	0.1	0.0	0.1	0.5	0.1	0.3	0.2	0.1	0	0.1	0.3	0.1
**R**	0	0	0	0	0	0	0	0	0	4	0	0	10	10	0	0
**S**	10	10	10	10	10	10	10	10	10	6	10	10	0	0	10	10

*Resistance.

CAZ, Ceftazidime; CFL, Cephalexin; CFM, Cefixime; CPD, Cefpodoxime; CRO, Ceftriaxone; CTX, Cefotaxime; CXM, Cefuroxime; FEP, Cefepime; P, *Pseudomonas aeruginosa*; PD, prepared disc; R, resistant; RP, resistant to prepared antibiotics; RS, pesistant to standard antibiotics; S, susceptible; SD, standard disc.

**Table 8.** Susceptibility of *Salmonella* sp. to commonly prescribed cephalosporins (standard and laboratory prepared disc)

	CFL (*P*<0.05)		CXM (*P*>0.05)		CFM (*P*>0.05)		CPD (*P*<0.05)		CRO (*P*<0.05)		CAZ (*P*<0.05)		CTX (*P*<0.05)		FEP (*P*<0.05)	
	SD	PD	SD	PD	SD	PD	SD	PD	SD	PD	SD	PD	SD	PD	SD	PD
**S1**	19±	16±	18±	17±	22±	22±	24±	23±	22±	20±0	20±	19±0	23±0*	22±*	25±	24±
	0.2	0.2	0.1	0.1	0.3	0.5	0.5	0.5	0.0	0.0	0.3	0.3	3	0.1	0.1	0.1
**S2**	18±	15±	17±	14±*	20±	19±	22±	21±	23±	22±0	21±	18±0	22±0*	21±*	24±	23±
	0.2	0.4	0.5	0.2	0.5	0.1	0.5	0.1	0.2	0.2	0.5	0.0	0	0.5	0.2	0.2
**S3**	18±	15±	20±	19±	20±	17±*	22±	21±	22±	20±0	20±	19±0	22±0*	21±*	25±	23±
	0.1	0.3	0.5	0.1	0.1	0.1	0.1	0.0	0.3	0.1	0.4	0.1	1	0.2	0.1	0.1
**S4**	18±	14±*	20±	19±	20±	19±	23±	21±	22±	21±0	21±	19±0	22±0*	20±*	26±	25±
	0.1	0.1	0.7	0.1	0.2	0.5	0.5	0.4	0.2	0.3	0.1	0.1	5	0.0	0.4	0.1
**S5**	14±*	14±*	18±	17±	21±	20±	23±	21±	20±	19±0*	23±	22±0	26±0.	24±	24±	23±
	0.3	0.2	0.1	0.1	0.3	0.1	0.1	0.1	0.2	0.1	0.3	0.2	5	0.1	0.6	0.5
**S6**	19±	16±	17±	14±*	21±	20±	21±	20±	19±*	17±0*	23±	22±0	25±0.	24±	24±	23±
	0.1	0.5	0.1	0.5	0.7	0.7	0.2	0.2	0.1	0.5	0.5	0.2	2	0.0	0.5	0.5
**S7**	19±	15±	17±	16±	21±	19±	24±	23±	19±*	18±0*	24±	22±0	24±0.	24±	24±	23±
	0.2	0.4	0.6	0.1	0.0	0.2	0.5	0.2	0.4	0.2	0.3	0.3	1	0.4	0.1	0.1
**S8**	19±	17±	20±	19±	20±	18±	24±	23±	23±	20±0	22±	21±0	24±0.	23±*	26±	25±
	0.6	0.2	0.2	0.2	0.4	0.2	0.2	0.3	0.5	0.1	0.3	0.4	1	0.1	0.2	0.1
**S9**	18±	14±*	18±	17±	22±	21±	24±	22±	22±	21±0	24±	23±0	26±0.	25±	26±	25±
	0.2	0.2	0.5	0.2	0.2	0.4	0.5	0.6	0.1	0.3	0.5	0.7	4	0.5	0.7	0.3
**S1**	14±*	14±	18±	17±	22±	22±	23±	21±	20±	19±0*	22±	21±0	26±0.	24±	25±	24±
**0**	0.1	0.1	0.5	0.7	0.1	0.0	0.5	0.1	0.9	0.0	0.1	0.1	0	0.1	0.7	0.1
**R**	2	4	0	2	0	1	0	0	2	4	0	0	4	5	0	0
**Sp**	8	6	10	8	10	9	10	10	8	6	10	10	6	5	10	10

*Resistance.

CAZ, Ceftazidime; CFL, Cephalexin; CFM, Cefixime; CPD, Cefpodoxime; CRO, Ceftriaxone; CTX, Cefotaxime; CXM, Cefuroxime; FEP, Cefepime; PD, prepared disc; R, resistant; RP, resistant to prepared antibiotics; RS, resistant to standard antibiotics; S, *Salmonella* sp.; SD, standard disc; Sp, susceptible.

However, Santosh *et al*. [26] reported an antibiotic susceptibility pattern of *Staphylococcus aureus*, *E. coli*, *P. aeruginosa* and * K. pneumoniae* to ceftazidime, ceftriaxone and cefoxitin as having a higher activity against these isolates. However, this result differs from the findings of Nwankwo *et al*. [27], who reported less activity of cephalosporin antibiotics such as cefuroxime, ceftazidime, ceftriaxone, cefotaxime and cefepime when tested against *P. aeruginosa*, *K. pneumoniae*, *Staphylococcus aureus* and * E. coli.* Among the Gram-negative isolates, resistance by *E. coli* and *K. pneumoniae* was higher. A similar pattern has been reported from Pakistan by Zubair *et al*. [28] and Jombo *et al*. [29] from Calabar, Nigeria.

The summary of the susceptibility of the tested organisms to standard cephalosporin antibiotics (Table 9) and that of the branded cephalosporin antibiotics (Table 10) identified as the most commonly sold shows that cefepime, a fourth-generation cephalosporin, exerts 100% activity to all of the tested organisms in both the standard and branded cephalosporins. However, cefotaxime (60%) and ceftriaxone (80%), third-generation cephalosporins, recorded the highest number of resistance to all the tested isolates in the standard antibiotics. A similar pattern of resistance was also observed in the branded cefotaxime and ceftriaxone antibiotics. A number of different mechanisms might be responsible for the decreased susceptibility of the antibiotics such as the presence of CTX-M genes in *E. coli* and *K. pneumoniae*, which confer resistance to third-generation cephalosporins like cefotaxime and ceftriaxone, alteration in the penicillin-binding proteins, reduction in the antibiotic penetration power, the action of *β*-lactamases and extended spectrum *β*-lactamases, as well as enhanced efflux pump mechanisms [30,32]. The absence of activity of cefotaxime against *P. aeruginosa* in Table 9, where 0% susceptibility was recorded, aligns with the known intrinsic resistance of the isolate to several third-generation cephalosporins, including cefotaxime. This resistance is largely due to the low permeability of its outer membrane, the presence of efflux pumps and the production of inducible AmpC *β*-lactamases, which hydrolyse cefotaxime and render it ineffective [31]. In clinical practice, *P. aeruginosa* infections are typically treated with other antipseudomonal agents such as ceftazidime, cefepime, piperacillin–tazobactam or carbapenems, rather than cefotaxime [31,32]. The results in Table 9, thus, reflect the expected pharmacodynamic profile and resistance mechanisms of *P. aeruginosa* [33*,* 34*],* confirming the accuracy and reliability of our susceptibility testing.

**Table 9.** Summary of the susceptibility of the tested organisms to standard cephalosporin antibiotics

Organisms	Cfl (%)	Cxm (%)	Cfm (%)	Cpd (%)	Cro (%)	Caz (%)	Ctx (%)	Fep (%)
*E. coli*	9 (90)	8 (80)	8 (80)	9 (90)	8 (80)	10	7 (70)	10 (100)
*K. pneumoniae*	10 (100)	9 (90)	8 (80)	10 (100)	8 (80)	9 (90)	7(70)	10 (100)
*Staphylococcus aureus*	10 (100)	10 (100)	10 (100)	10 (100)	10 (100)	10 (100)	10 (100)	10 (100)
*Streptococcus pneumoniae*	10 (100)	10 (100)	10 (100)	10 (100)	10 (100)	10 (100)	8 (80)	10 (100)
*P. aeruginosa*	10 (100)	10 (100)	10 (100)	10 (100)	10 (100)	10 (100)	0	10 (100)
*Salmonella* spp.	8 (80)	10 (100)	10 (100)	10 (100)	8 (80)	10 (100)	6 (60)	10 (100)

Caz, Ceftazidime; Cfl, Cephalexin; Cfm, Cefixime; Cpd, Cefpodoxime; Cro, Ceftriaxone; Ctx, Cefotaxime; Cxm, Cefuroxime; Fep, Cefepime.

**Table 10.** Summary of the susceptibility of the tested organisms to branded cephalosporin antibiotics

Organisms	Cfl (%)	Cxm (%)	Cfm (%)	Cpd (%)	Cro (%)	Caz (%)	Ctx (%)	Fep (%)
*E. coli*	9 (90)	8 (80)	8 (80)	8 (80)	7 (70)	8 (80)	5 (50)	10 (100)
*K. pneumoniae*	8 (80)	8 (80)	8 (80)	8 (80)	6 (60)	7 (70)	6 (60)	10 (100)
*Staphylococcus aureus*	10 (100)	9 (90)	9 (90)	10 (100)	7 (70)	8 (80)	7 (70)	10 (100)
*Streptococcus pneumoniae*	10 (100)	10 (100)	8 (80)	10 (100)	8 (80)	8 (80)	6 (60)	10 (100)
*P. aeruginosa*	10 (100)	10 (100)	10 (100)	10 (100)	6 (60)	10 (100)	0	10 (100)
*Salmonella* sp.	6 (60)	8 (80)	9 (90)	10 (100)	6 (60)	10 (100)	5 (50)	10 (100)

Caz, Ceftazidime; Cfl, Cephalexin; Cfm, Cefixime; Cpd, Cefpodoxime; Cro, Ceftriaxone; Ctx, Cefotaxime; Cxm, Cefuroxime; Fep, Cefepime.

FT-IR analysis of the purchased antibiotics (cephalexin, cefuroxime, cefixime and cefpodoxime antibiotics) (Table 11) revealed the presence of alcohol (OH) group, carboxylic acid, alkane and alkyne. Aromatic group (benzene) was observed in the cephalexin antibiotic. Sharp peaks observed at lower band gap grouping from 1,119 to 1,992 correspond to the sulphide presence in a compound. Thus, the observed peaks correspond to the molecular formula of the compound cephalexin (Appendix III). In the cefuroxime antibiotic, compounds such as amides N-H, alkane C-H, NH2 (amines) and alkene (C=C), esters, carboxylic acid, alkene and cyanite were observed to be present which corresponds to the molecular formula of the compound cefuroxime (Appendix IV). The presence of amides (N-H) and alcohol (OH) shows the presence of alkene and alkane. The peaks confirm the presence of aldehyde (CHO), cyano (C-N), aromatics and aldehyde C-O, respectively. This corresponds with the formula of the compound cefixime (Appendix V). The peaks confirm the presence of N-H (amides), OH (alcohol), C═C-H (alkene), alkene and aldehyde. This corresponds with the formula of the compound cefpodoxime (Appendix VI). The presence of OH (alcohol), amide (NH2) and amide (NH), alkane (C-H), cyanite (C=N), aldehyde (CHO), C-N) and aromatics (Table 12) was confirmed, thus corresponding with the formula of ceftriaxone (Appendix VII). In the analysis of the suspected ceftazidime antibiotic, the presence of OH (alcohol), C-O, C=C, C-N and C-H at the various peaks of 1,250, 2,117, 1,500 and 1,566 was also noted. The strong peaks at C=N, C-N, aromatics and N-O stretching were seen at peaks 1,591, 1,540, 1,600 and 1,382, respectively. The peak at low brand corresponds to sulphide, and thus, the formula is suited to the antibiotic ceftazidime (Appendix VIII). The presence of C═C-H alkene group, NH2 amines, C-H alkanes and alkenes was observed. Sharp peak at 1,735–1,750 confirmed RCOOR esters. This confirms the presence of cefotaxime (Appendix IX). The presence of N-H (amides), OH (alcohol) and CC-H (alkene) was observed. Sharp peak at 1,778 confirmed C=O (carbonyl), while at 1,720, 1,250 and 905 showed CHO (aldehyde) C-O (aldehyde), C=O (carbonyl) and S (sulphide), respectively, corresponding to the formula cefepime (Appendix X). This analysis of the commercially obtained cephalosporins confirmed the presence of key functional groups consistent with the expected molecular structures of each antibiotic, as documented in pharmacopeial references. These findings indicate that the antibiotics tested were chemically intact and structurally aligned with their respective formulations. No missing or anomalous functional groups were observed that would suggest substandard or counterfeit products. Although certified reference standards were not analysed alongside, the consistency of the spectral data with established profiles supports the pharmaceutical authenticity and bioavailability of the antibiotics studied.

**Table 11.** FT-IR analysis of cephalexin, cefuroxime, cefixime and cefpodoxime antibiotics

Antibiotics	Peaks cm^−1^	Compound group	Molecular structure
Cephalexin	3,662	OH (alcohol)	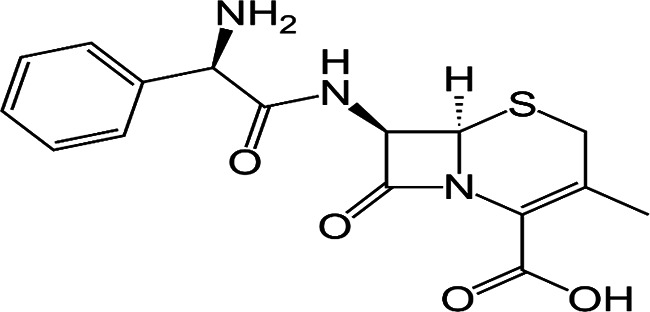
	3,382	O═C-OH (carboxylic acid)	
	2,975	C-H (alkane)	
	2,100	C≡C (alkyne)	
	1,760	Aromatic	
	1,886	C═C alkyl	
	1,480	C═N nitrile	
	1,760	C═O carbonyl	
	1,119−900	S (sulphide)	
Cefuroxime	3,562	N-H	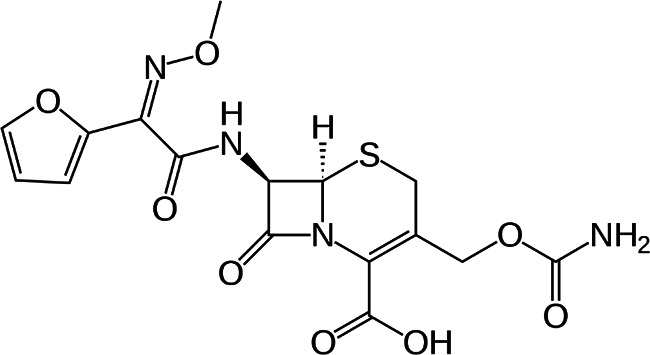
	3,324	C-H	
	2,940	NH2	
	2,121	C═C	
	1,994	C═O	
	1,547	Aromatics	
	1,787	Esters	
	1,787	Oll R-C-OH (carboxylic acid)	
	1,670	C═C	
	1,428	C═N	
	855	S (sulphide)	
	1,164	C-O	
	1,376	N-O	
Cefixime	3,284	NH_2_ (amines)	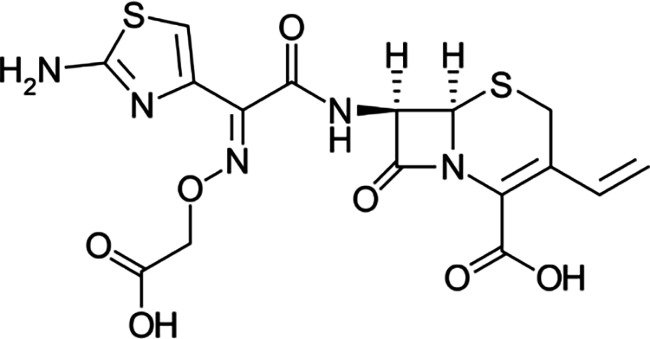
	1,771	-C═O (carboxylic acid)	
	1,667	N-H (amide)	
	1,540	C-H (alkane)	
	1,591	C═N (cyanite)	
	1,382	N-O (stretching)	
	1,669	C═O (amide)	
Cefpodoxime	3,582	N-H (amides)	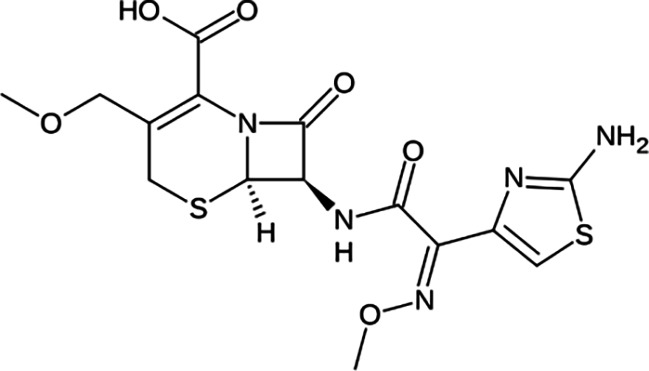
	3,387	OH (alcohol)	
	3,324	C═C-H (alkene)	
	2,998	C-H (alkane)	
	1,644	Aromatics	
	2,125	C═C (alkene)	
	1,763	CHO (aldehyde)	
	1,238	C-N (cyano)	
	1,250	C-O (aldehyde)	
	992	Sulphide	

**Table 12.** FT-IR analysis of ceftriaxone, ceftazidime, cefotaxime and cefepime antibiotics

Antibiotics	Peaks cm^−1^	Compound group	Molecular structure
**Ceftriaxone**	3,655.8	OH (alcohol)	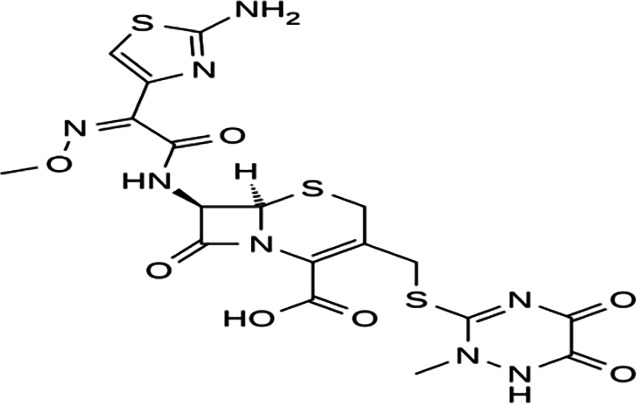
	3,551.9	NH (amides)	
	3,280.8	NH_2_ (amines)	
	2,936.8	C-H (alkane)	
	2,200	C≡N (nitriles)	
	2,100	C≡C (alkyne)	
	1,740	CHO (aldehyde)	
	1,365	N-O	
	1,033	C-O (aldehyde)	
	1,698	C═N (cyanite)	
	1,238	C-N (cyano)	
	1,652	Benzene	
**Ceftazidime**	3,238	OH (alcohol)	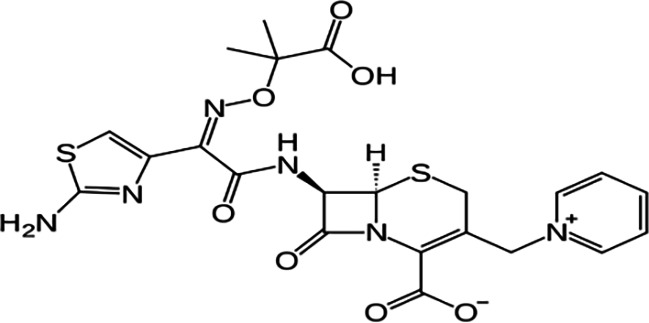
	1,250	C-O (aldehyde)	
	2,117	C≡C (alkyne)	
	1,500	C-N (cyano)	
	1,566	C-H (alkane)	
	1,382	N-O	
	1,591	C═N (cyanite)	
	1,540	C-H (alkane)	
	1,600	Benzene	
**Cefotaxime**	3,650–3,200	OH group	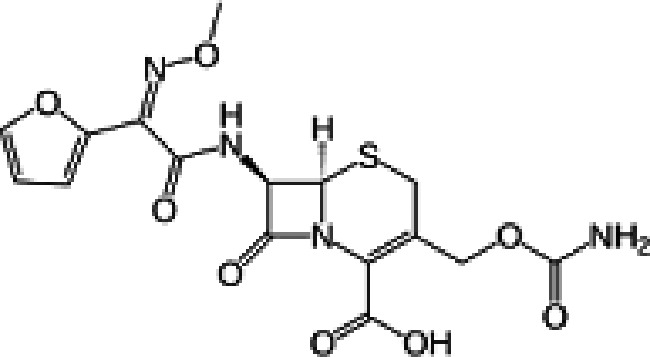
	3,020–3,100	C═C-H alkene group	
	3,300–3,350	NH2 amines	
	2,850–2,975	C-H alkane	
	2,100–2,250	C═C alkyne	
	1,430–1,500	Aromatics	
	1,735–1,750	RCOOR esters	
	1,230–1,020	C═C alkene	
	1,248	CO aldehyde	
**Cefepime**	3,500	N-H (amide)	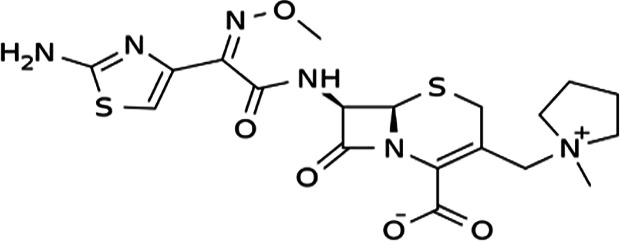
	3,400	O-H (alcohol)	
	3,300	C═C-H (alkenes)	
	1,778	C═O (carbonyls)	
	1,720	CHO (aldehyde)	
	1,250	C-O (aldehyde)	
	905	S (sulphide)	

**Fig. 1.**
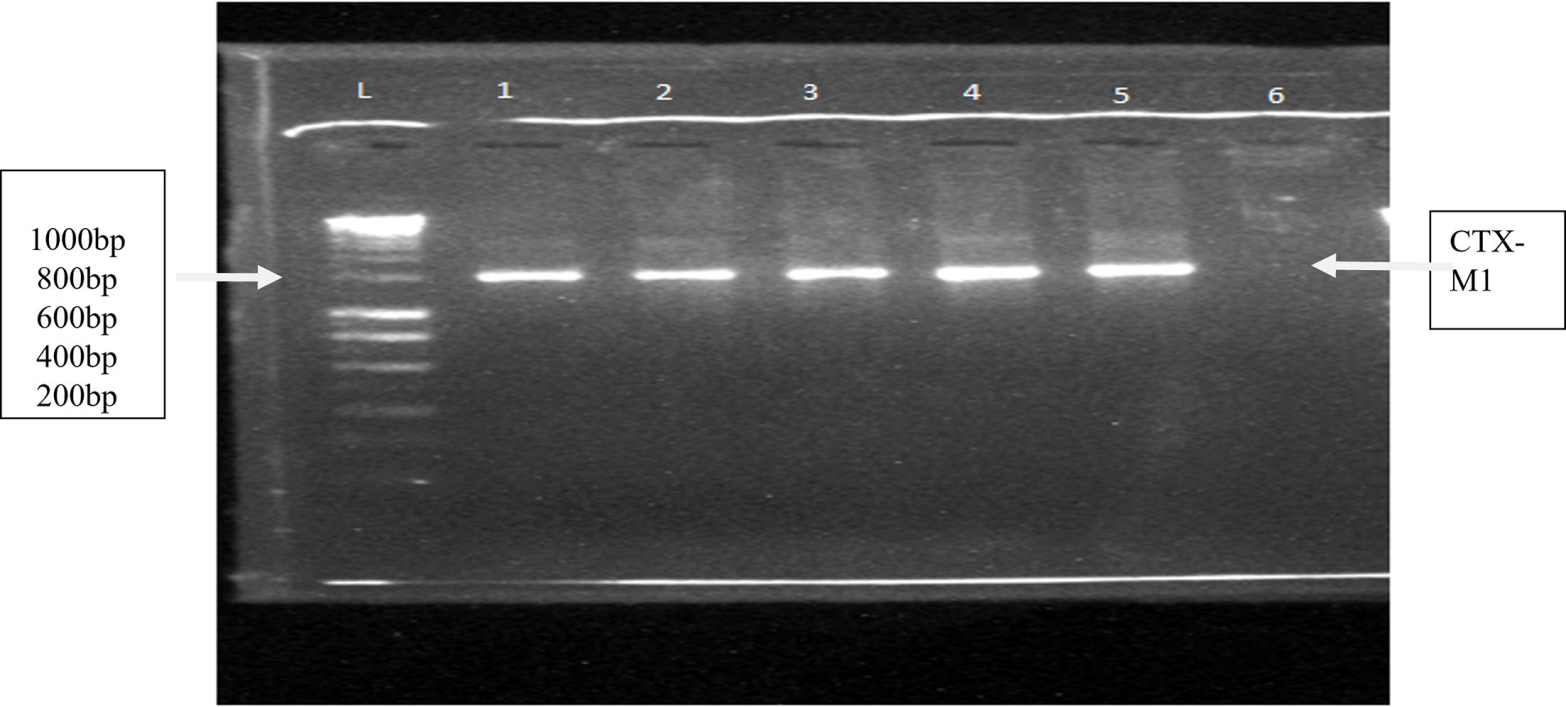
Agarose gel electrophoregram. L indicates DNA ladder (Hyperladder). Lane 1, E4; lane 2, E7; lane 3, K3; lane 4, K7. Lane 5: positive control. Lane 6: negative control. All tested isolates (two *E. coli* ‘E4 and E7’ and two *K. pneumoniae* ‘K3 and K7’) were positive for the CTX-M1 resistance gene.

The most resistant isolates were selected for molecular identification of the resistance gene (Fig. 1). A total of four isolates, two from *E. coli* (E4 and E7) and two from *K. pneumoniae* (K3 and K7), were used for the detection of the CTX-M1 gene. All isolates tested positive for the CTX-M1 gene.

## Conclusion

This study assessed the antimicrobial susceptibility patterns of clinically important bacteria to commonly used cephalosporins obtained from Sabon Gari Market, Kano. While most branded antibiotics exhibited comparable activity to standard formulations, resistance to third-generation cephalosporins, particularly cefotaxime and ceftriaxone, was observed in 20% of *E. coli* and *K. pneumonia*e isolates. Four of these resistant strains (two *E. coli* and two *K. pneumoniae*) were confirmed to harbour the CTX-M1 gene. FT-IR analysis validated the presence of characteristic functional groups in all tested antibiotics, indicating structural integrity and pharmaceutical quality. Overall, the findings suggest that while the cephalosporins tested are largely pharmaceutically sound, there is an emerging resistance in some bacterial strains that warrants continuous surveillance.

## Recommendation

- Determination of the antibacterial efficacy of the most commonly used cephalosporin antibiotics should be conducted regularly.
- Proper handling of antibiotics, as well as dose completion, should be encouraged as this is linked with the growing level of microbial multidrug resistance.
- Detection of other resistant genes should be conducted as a particular isolate may harbour many different resistant genes.
- FT-IR analysis should be employed to confirm the functional group of the active ingredients of other most commonly used antibiotics, other than cephalosporins.

## Supplementary material

10.1099/acmi.0.000837.v4 Uncited Supplementary Material 1.

## Abbreviations

AMR: antimicrobial resistance
API: active pharmaceutical ingredient
AR: antibiotic resistance
CLSI: Clinical and Laboratory Standards Institute
FT-IR: Fourier transform infrared spectroscopy
NAFDAC: National Agency for Food and Drug Administration and Control
TSI: triple sugar iron
