# Supplementary material for: Evaluation of the efficacy of cephalosporin antibiotics sold in Kano, Nigeria, against clinical bacterial isolates
Source: Access Microbiol. 2025 Jul 14;7(7):000837.v4. doi: 10.1099/acmi.0.000837.v4 (PMC12258513; doi:10.1099/acmi.0.000837.v4)

Kano State APPENDICES

Appendix i: Ethical Approval

KANO STATE OF NIGERIA
MINISTRY OF HEALTH
2nd & 3rd Floor, Post Office Road,
P.M.B. 3066, Kano.

Hon.. Commissioner: 08039472476
Permanent Secretary: 08023307354
Email: moh.kano2019@gmail
Website: www.smogorg.ng/webmail

Ref: SHREC/2021 /2406 *Date:* 18th May, 2021

NHREC Approval Number; NHREC/17/03//2018

Safiyya Ibrahim Atiku,
Department of Microbiology,
Faculty of Life Science,
College of Health Sciences,
Bayero University,
Kano.

RE: APPLICATION FOR ETHICAL APPROVAL

Reference to your letter dated 10th May, 2021 on the above request addressed to the Chairman Health Research Ethics Committee of the Ministry requesting for ethical approval to conduct a Research work at Bayero University, Kano State.

2. The research entitled ***"Evaluation of Susceptibility Status of Some Bacterial Clinical Isolates to Generations of Cephalosporin's Sold in Kano"*** is for the Award of Master of Science Degree in Microbiology.

3. In view of the foregoing, I wish to convey the Ministry's approval for you to conduct the research at the above mentioned Institution.

4. You are also requested to share your findings with the Ministry of Health, Kano state.

5. Best Regards

Jibrin Habu
DPRS
Secretary (HREC)
For: Honourable Commissioner

Appendix ii: Questionnaire

Knowledge about the MOST Commonly Sold Cephalosporins Antibiotics In Kano

Tick the appropriate Answer

1) i) **First Generation Cephalosporins Most Commonly Sold**

a) Cephalexin
b) Cepharadine
c) Cefadioxil
d) Cefazolin

1ii) why? Because it is…

a) Cheap
b) Effective
c) Available
d) Prescribed by medical practictioner

2i) Second Generation Cephalosporins Most Commonly Sold

a) Cefoxitin
b) Cefotetan
c) Cefroxil
d) Cefuroxime
e) Cefaclor

2ii) why? Because it is…..

a) Cheap
b) Effective
c) Available
d) Prescribed by Medical Practioner

3i).Third Generation Cephalosrins Most Commonly Sold

a) Cefpodoxime
b) Ceftszidime
c) Ceftributen
d) Cefotaxime
e) Ceftazidime
f) Ceftriaxone
g) Ceftrixime
h) Cefdinie
i) Cefixime

3ii) Why? Because it is….

a) Cheap
b) Effective
c) Available
d) Prescribed by Medical Practioner

4i) Fourth Generation Cephalosporinsn Most Commonly Sold

a) Ceftararoline
b) Cefepime

4ii)why? Because it is….

a) Cheap
b) Effective
c) Available
d) Prescribed by Medical Practioners

Appendix iii: FT-IR Chromatogram of Cephalexin antibiotic

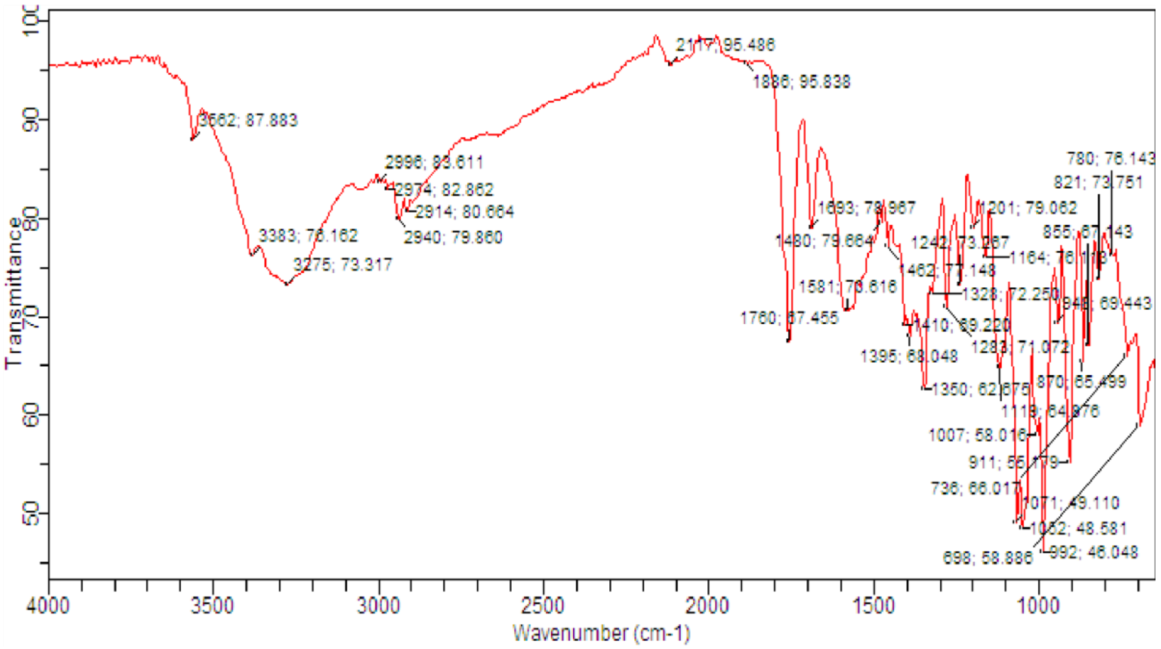

Appendix iv: FT-IR Chromatogram of Cefuroxime antibiotic

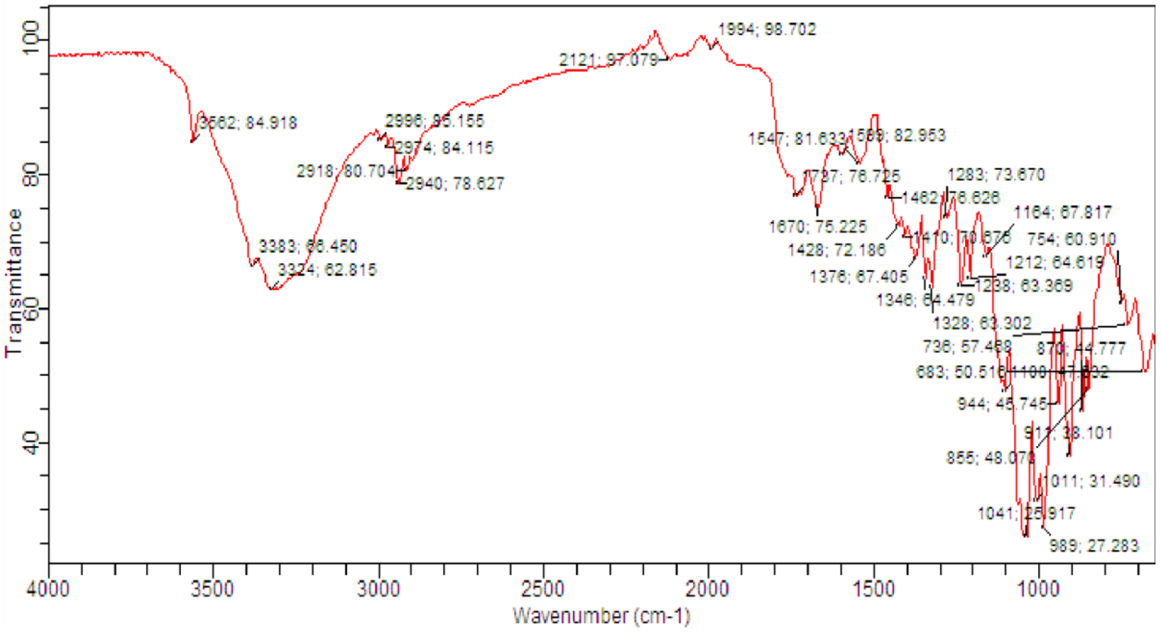

Appendix v: FT-IR Chromatogram of Cefexime antibiotic

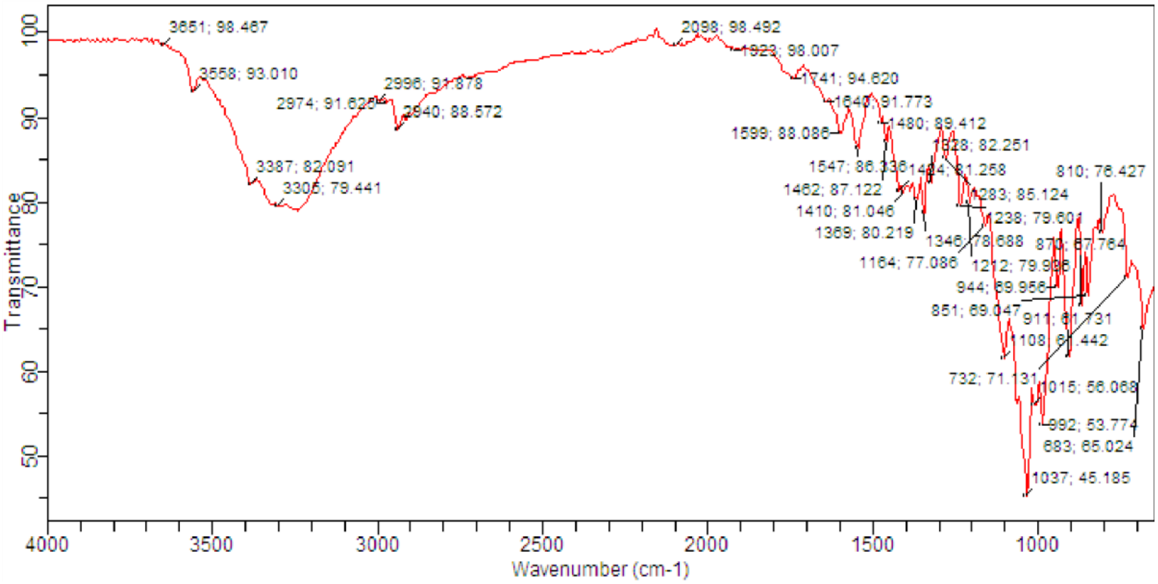

Appendix vi: FT-IR Chromatogram of Cefpodoxime antibiotic

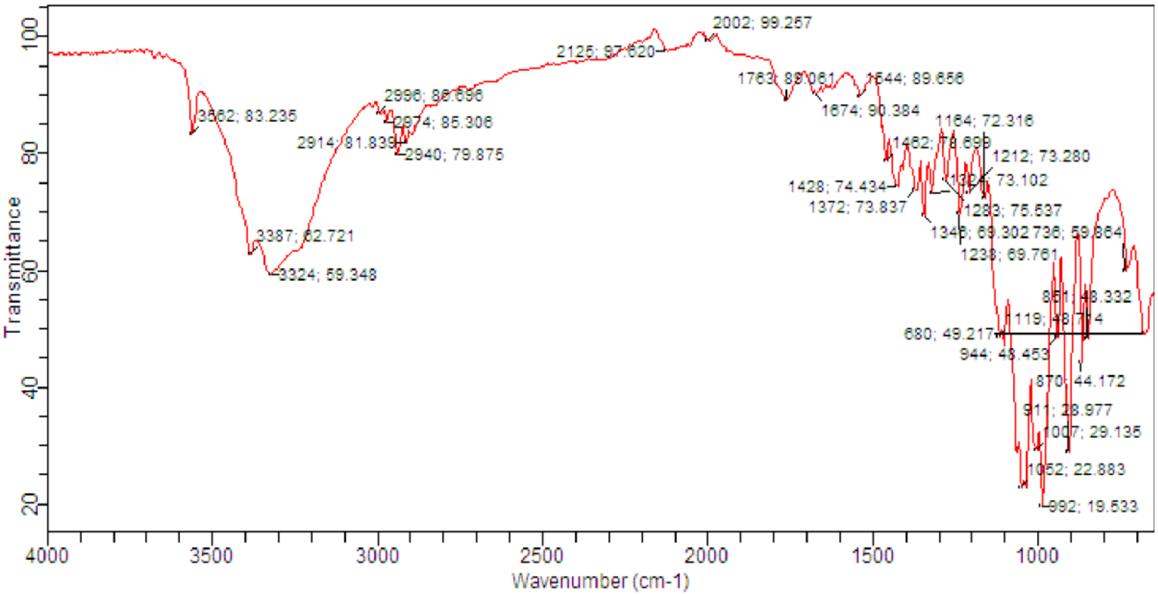

Appendix vii: FT-IR Chromatogram of Ceftriazone antibiotic

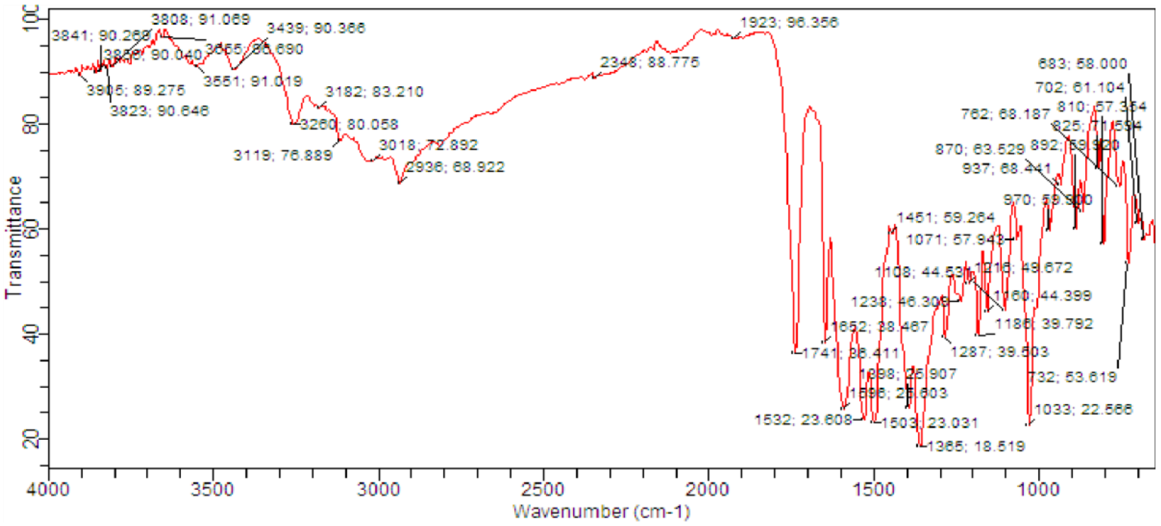

Appendix viii: FT-IR Chromatogram of Ceftazidime antibiotic

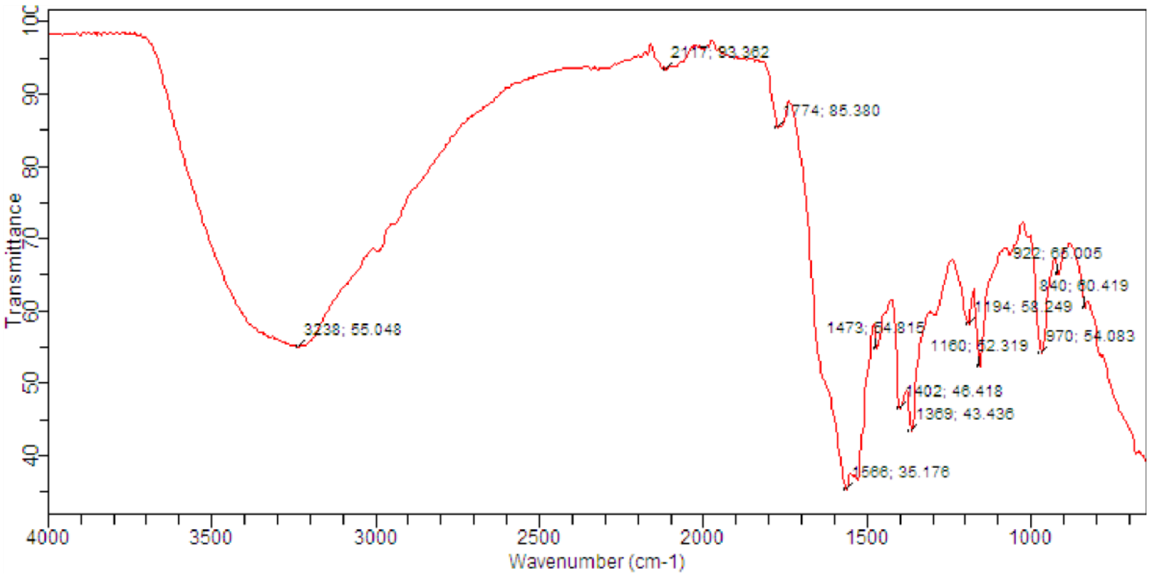

Appendix ix: FT-IR Chromatogram of Cefotaxime antibiotic

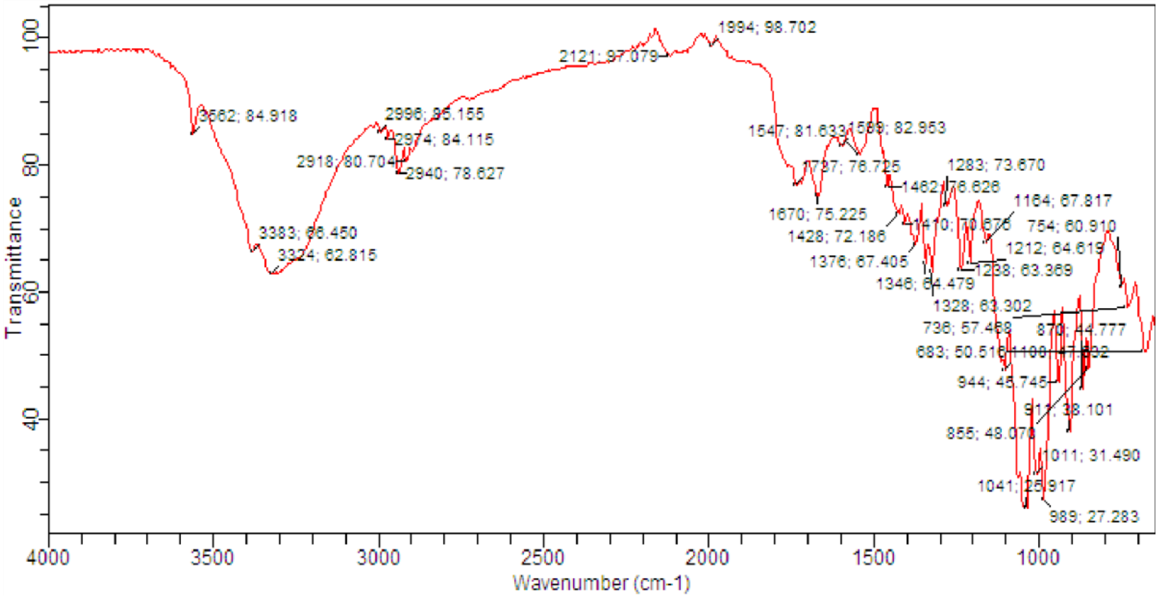

Appendix x: FT-IR Chromatogram of Cefepime antibiotic

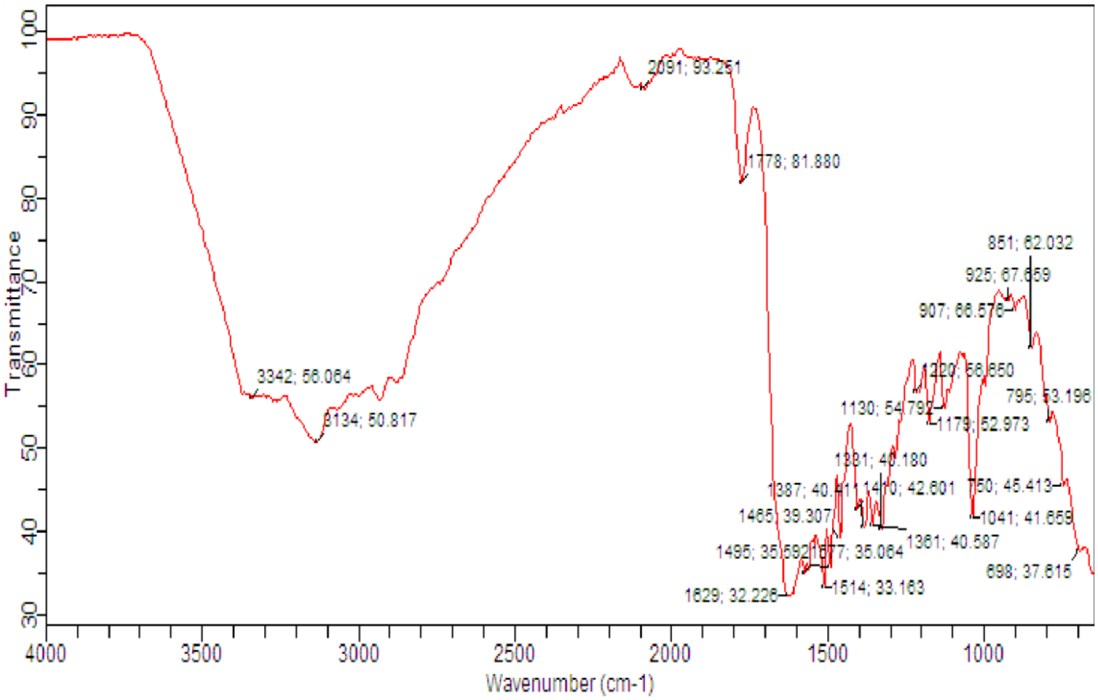

Supplement: Uncited Supplementary Material 1. [file acmi-7-00837-s001.pdf]
